# Microbiological Profiles of Patients with Periprosthetic Joint Infection of the Hip or Knee

**DOI:** 10.3390/diagnostics12071654

**Published:** 2022-07-07

**Authors:** Frank Sebastian Fröschen, Thomas Martin Randau, Alexander Franz, Ernst Molitor, Gunnar Thorben Rembert Hischebeth

**Affiliations:** 1 Department of Orthopaedics and Trauma Surgery, University Hospital Bonn, 53127 Bonn, Germany; thomas.randau@ukbonn.de (T.M.R.); alexander.franz@ukbonn.de (A.F.); 2 Institute of Medical Microbiology, Immunology and Parasitology, University Hospital Bonn, 53127 Bonn, Germany; molitor@uni-bonn.de (E.M.); hischebeth@microbiology-bonn.de (G.T.R.H.)

**Keywords:** hip, knee, periprosthetic joint infection, antimicrobial resistance, microorganism

## Abstract

Periprosthetic joint infections (PJI) are one of the most devastating consequences after total joint arthroplasty. We sought to analyze the causative pathogens of patients with PJI to get better insights and improve treatment. We performed a retrospective study of all patients with PJI of the hip and knee with microbiological detection of a causative pathogen at a tertiary endoprothetic referral center between January 2016 and March 2021. A total of 432 cases with PJI (hip: *n* = 250; knee: *n* = 182) were included. The most common causative pathogen were coagulase-negative staphylococci (*n* = 240; 44.2%), of which *Staphylococcus epidermidis* (*n* = 144; 26.7%) was the most frequently detected, followed by *S. aureus* (*n* = 77; 14.3%) and enterococci (*n* = 49; 9%). Gram-negative pathogens and fungi could be detected in 21% (*n* = 136) and 2.4% (*n* = 13) of all cases. Overall, 60% of all coagulase-negative staphylococci were oxacillin-resistant, while none of these displayed to be vancomycin-resistant. In summary, the majority of pathogens in cases of PJI could be identified as coagulase-negative staphylococci. For empirical therapy vancomycin might provide the highest antimicrobial coverage in case of an unknown pathogen.

## 1. Introduction

Total joint arthroplasty (TJA) of the hip or knee is still one of the most successful orthopedic treatment options in patients with need for arthroplasty. Periprosthetic joint infection (PJI) is a devastating complication of arthroplasty [1]. Although the incidence of PJI after total hip arthroplasty (THA) or total knee arthroplasty (TKA) is low and described between 1–2% for primary arthroplasties with higher rates of up to 20% in revision arthroplasty [2,3,4]. With rising numbers of arthroplasties performed, a subsequent increase of patients with PJI has to be considered [5]. 

Previous studies could already outline that different causative pathogens affect the outcome [6,7,8,9]. For treatment success, a knowledge of the local microbiological profile and antimicrobial resistance data are helpful. However, the prevalence of causative pathogens and their antibiotic resistances may vary. Often, the infectious organism has not been identified at the start of the antimicrobial therapy. In these cases, local epidemiological data of PJIs may be helpful. These data may be used to determine the best possible empiric antibiotic therapy. 

The purpose of this study was to characterize the causative pathogens for infection of the inlying total hip or total knee arthroplasties and to evaluate antibiotic susceptibility of the most common pathogens. 

## 2. Materials and Methods

In this retrospective study, we included all consecutive cases of PJI of the hip or knee joint at a tertiary endoprothetic referral center in Germany between January 2016 and March 2021. Inclusion criteria were presence of a PJI of the hip or knee joint with microbiological detection of a causative pathogen in specimens (intraoperative tissue biopsies, sonication and synovial fluid) obtained intraoperatively. 

Shredded and homogenized intraoperatively collected tissue specimen as well as sonication fluid (0.5 mL) were plated on Columbia agar with 5% sheep blood, MacConkey agar, chocolate agar, and sabouraud agar (Becton & Dickinson, Bergen County, NJ, USA), while 1 mL was pipetted into thioglycolate boullion (Becton & Dickinson, Bergen County, NJ, USA). 

For anaerobic cultures schaedler and kanamycin/vancomycin agar plates (Becton & Dickinson, Bergen County, NJ, USA) were struck with 0.5 mL sonication fluid or with shredded and homogenized tissue specimen. All cultures were grown at 5% CO_2_ and 35 °C for at least 14 days. In parallel, sonication fluid was added in PEDSmedium blood culture flasks (Becton & Dickinson, Bergen County, NJ, USA) and incubated in a Bactec FX blood culture system (Becton & Dickinson, Bergen County, NJ, USA) for 14 days. Joint aspirates were inoculated in PEDSmedium blood culture flasks (Becton & Dickinson, Bergen County, NJ, USA) and incubated in a Bactec FX blood culture system (Becton & Dickinson, Bergen County, NJ, USA) for 14 days.

The identification of pathogens was carried out using matrix-assisted LASER desorption/ionization time-of-flight (MALDI-TOF) spectroscopy (bioMérieux, Nürtingen, Germany). Additionally, the antimicrobial susceptibility testing was performed with an automated antimicrobial susceptibility testing system, Vitek2 (bioMérieux, Nürtingen, Germany). In the case of detection of anaerobic pathogens, susceptibility testing was carried out with a semiautomated microtiter broth dilution system (MICRONAUT; Merlin, Bornheim, Germany). For interpretation of antimicrobial susceptibility the EUCAST clinical breakpoints (v. 12.0, 2022) were applied.

A PJI was defined according to Parvizi et al. with fulfilling one of the following criteria: (1) a sinus tract communicating with the prosthesis, (2) isolation of the same microorganism from two or more cultures/tissue biopsies obtained from the infected joint or (3) isolation of one microorganism in the intraoperative cultures with additional evidence of an infection of the inlying implant (positive histology, presence of purulence, elevated serum erythrocyte sedimentation rate, elevated C-reactive protein and elevated synovial white blood cell count) [10].

For better description of the included patients, we recorded patient demographics, weight, site of arthroplasty, surgery time (cutting/suture), comorbidities, performed procedure and preoperative anemia. Based on the time interval between surgery and infection, we classified the infection as the following modified according to Izakovicova et al. [4]: acute early onset (occurring within 6 weeks after surgery with symptom duration less than 3 weeks), and persisting early onset (occurring within 6 weeks after surgery with symptom duration more than 3 weeks; e.g., persisting infection after failed treatment of acute PJI), acute late onset (occurring after 6 weeks after surgery with symptom duration less than 3 weeks) and chronic late onset (occurring after 6 weeks after surgery with symptom duration more than 3 weeks). If during this study patients underwent surgery several times of the same joint only the first episode was recorded. 

A polymicrobiological PJI was defined as detection of more than one microorganism isolated from the intraoperative tissue biopsies, sonication or synovial fluid. The microbiological profiles of all pathogens were analyzed. 

### Statistical Analysis

Data were collected in Microsoft Excel 2022 (Microsoft Corporation, Richmond, VA, USA). Statistical analysis was carried out with SPSS statistics 28 for Windows (SPSS, Inc. an IBM company, Chicago, IL, USA). Descriptive statistics, including arithmetic mean value and standard deviation were calculated. Data are given as means ± standard deviation (SD) and ranges, if not indicated otherwise. 

To analyze categorial data Fisher’s exact test was used to test for an association (*p* < 0.05). In detail we used the Fisher’s exact test to test for an association between patients with PJI of the hip or knee and sex or comorbidities. For comparison of the age, BMI, preoperative creatinine and preoperative C-reactive protein of patients with PJI of the hip or knee joint a two-tailored *t* test was performed (*p* < 0.05). All statistics were two-sided. A Bonferroni correction for multiple comparisons was performed to adjust the *p*-value for final evaluation. 

## 3. Results

A total of 432 cases with PJI of the hip or the knee joint (hip: *n* = 250; knee: *n* = 182) were included between January 2016 and March 2021. The demographic data and comorbidities of all patients are presented in Table 1. 

No included patient had a bilateral joint infection. There was no difference in age or sex between patients with PJI of the knee or hip joint. Patients with TKA had a higher proportion of diabetes mellitus, immunosuppression and rheumatoid arthritis than patients with PJI of the hip. Patients with THA-PJI had a higher preoperative C-reactive protein than patients with PJI of the knee. After performing a Bonferroni correction for multiple comparisons with adjustment of the *p*-value, none of these were significant. Overall, 109 patients had an infection of the right hip, while 141 had an infection of the left hip. Additionally, 87 patients had an infection of the right knee, while 95 had an infection of the left knee. Most of the patients presented themselves with a chronic late PJI of the hip (60%) or knee (64%) joint. Mean surgery time of patients with PJI of the hip was 161.75 ± 73.78 min (Debridement, antibiotics, implant retention [DAIR]: 134.84 ± 56,76 min; Explantation of the inlying implant: 176.13 ± 77.85 min) and 146.71 ± 64.93 min (DAIR: 119.17 ± 58.25 min; Explantation of the inlying implant: 167.58 ± 62.1 min) in case of a PJI of the knee joint. 

Overall, 51 out of 250 Patients (20.4%) had a polymicrobial PJI of the hip, while 34 of 182 patients (18.7%) had a polymicrobial infection of the knee. Therefore, a monomicrobial infection could be detected in 347 of 432 cases (80.3%). In 4 cases with PJI of the hip 3 different pathogens (knee: 5 cases) could be detected, while in 4 cases with PJI of the hip 4 different pathogens (knee: 1 case) could be detected. We could not detect more than 4 different pathogens in any of the evaluated cases. In summary, we could detect 538 different pathogens in the 432 evaluated cases. 

As shown in Table 2, the most frequent pathogen were coagulase-negative staphylococci, which could be detected in 44.61% of the cases (hip: 48.56%, knee: 39.11%), followed by *S. aureus* (total: 14.31%; hip: 12.78%, knee 16.44%) and enterococci (total: 9.01%; hip: 8.95%; knee: 9.33%). Sub-group analysis of the coagulase-negative staphylococci revealed that *S. epidermidis* was the most frequent detected pathogen (total: 144 cases (26.7%); hip: 88 cases (28.11%); knee: 56 cases (24.89%)), followed by *S. haemolyticus* (total: 22 cases (4.1%); hip 15 cases (4.79%); knee: 7 cases (3.1%)) and *S. lugdunensis* (total: 20 cases (3.71%); hip: 9 cases (2.54%); knee: 11 cases (4.88%)). 

The microorganisms found in patients with polymicrobial infections are presented in Table 3. The most frequent pathogens were coagulase-negative *staphylococci*, which could be detected in 83.53% of all cases (hip: 92.16%; knee: 70.59%). In detail, *S. epidermidis* (49.41%) was the most frequently detect microorganism in cases with polymicrobial PJI of the hip or knee, followed by *E. faecalis* (23.53%) and *S. aureus* (22.35%). In cases with PJI of the hip, the most frequently detected Gram-negative microorganism was *P. mirabilis* (8.24%), while in patients with PJI of the knee *K. pneumonie* (11.76%) and *E. coli* (8.82%) could be detected more often. 

To evaluate the most frequent combinations of patients with polymicrobial PJI subgroup analysis was performed. The overall most frequent combination consisted of *S. aureus* with *E. faecalis* (4 cases), for patients with PJI of the hip the most frequent combination consisted of *E. faecalis* and *P. mirabilis* (3 cases), while in patients with PJI of the knee the combination of *E. faecalis* and *E. faecium* could be detected in three cases. 

In summary, the overall distribution of causative pathogens for patients with PJI of the hip or knee joint revealed that the most common isolated pathogen were aerobic Gram-positive bacteria (77.8%), followed by rod shaped Gram-negative bacteria (21.73%) and rod-shaped/anaerobic Gram-positive bacteria (6.13%). The proportion of these pathogens in patients with PJI of the hip and knee joint were similar for aerobic Gram-positive bacteria (hip 77%; knee: 79.1%), rod-shaped Gram-negative bacteria (hip: 13.74%; knee: 7.99%) and rod-shaped or anaerobic Gram-positive (hip: 6.07%; knee: 6.22%). 

The detection of *Candida* species as causative pathogen was possible in 13 cases (hip: 8; knee: 5). *C. albicans* was found in 9 cases (hip: 4; knee: 5), while *C. glabrata* (1 case), *C. tropicalis* (1 case), and *C. parapsilosis* (2 cases) were only found in cases with PJI of the hip. In cases with polymicrobial PJI, *Candida* species was found in four cases as coinfection with *E. faecalis*, *E. faecium*, *E. cloacae* and *S. haemolyticus*.

To evaluate antibiotic susceptibility against certain antibiotics, a sub-group analysis was performed as shown in Table 4. The majority of *S. aureus* showed susceptibility to oxacillin (93%) and rifampicin (93%), whereas the majority of the coagulase-negative staphylococci displayed a resistance to oxacillin (60.2%), while being susceptible to rifampicin (74.3%) and vancomycin (100%). According to the time to infection, the proportion of oxacillin-resistant coagulase-negative staphylococci varied between 57.2–69.4% (acute early onset: 69.4%; acute late onset: 57.1%; chronic late onset: 61.4%). 

For enterococci, evaluation of the antibiotic susceptibility revealed a resistance against ampicillin in 22.4% of the isolates, while resistance against vancomycin could be detected in 4% of the isolates (*n* = 2, both *E. faecium*). These two isolates displayed a resistance against the ampicillin, too. Gram-negative microorganisms showed resistance against piperacillin/tazobactam in 18.7%, for ciprofloxacin in 9.4% or for sulfamethoxazole-trimethoprim in 27%. None of the analyzed isolates displayed a resistance against meropenem. 

## 4. Discussion

Despite continuous improvement and the routine use of perioperative antibiotics, dental screening procedures and perioperative guidelines for asepsis within the operation theatre in modern medicine, the overall incidence of PJI remains approximately between 0.3–2% [3,9,11]. Within this study, we present one of the largest cohorts of patients with PJI of the hip or knee joint in Germany from a single institution. We were able to identify the most common pathogens in patients with PJI of the hip and knee joint. These data might assist orthopedic surgeons during interdisciplinary treatment of a PJI, especially in case of a culture negative PJI. 

Although previous studies have reported *S. aureus* as most prevalent pathogen in hip and knee PJI with detection rates of up to 26% according to Tsai et al., we could not confirm these results [5]. In our presented study, *S. aureus* was detected in 14.3% of all cases, while only 7% of all *S. aureus* isolates were tested oxacillin-resistant. This is clearly in contrast to the work by Bjerke-Kroll et al., who reported oxacillin resistance in *S. aureus* isolates in nearly 24% [12]. Here, it is certainly undoubtful that *S. aureus* plays a relevant role in implant related as well as surgical side infections as previous studies by Oliveira et al. could outline [13]. Nevertheless, severe consequences have to be considered in case of detection of oxacillin-resistant *S. aureus* strains with a consecutive high risk for a persisting infection [14,15]. 

In accordance with the results of Bjerke-Kroll et al., who described the detection of coagulase-negative staphylococci in 39.9% of all cases, our most frequently detected pathogens were coagulase-negative staphylococci, which could be detected in 44.6% of all cases. In contrast to previously described rates of oxacillin-resistance between 22.6% reported by Bjerke-Kroll et al. or 26.8% reported by Peng et al., 60% of our coagulase-negative staphylococci displayed a oxacillin-resistance [12,16].

Some authors describe coagulase-negative staphylococci as frequently detected pathogens in case of a contamination of the intraoperative tissue biopsies, which is understandable as, e.g., *S. epidermidis* is a member of commensal skin flora [12,17]. In this context, Widerström et al. tried to evaluate the heterogeneity of *S. epidermidis* in PJI with the help of whole-genome sequencing. Their main finding was, that even *S. epidermidis* isolates taken only from patients with confirmed PJI displayed an astonishingly high ambiguity [18]. Although they were not able to distinguish between contamination and detection of the causative pathogen with whole-genome sequencing, they suggested that detection of *S. epidermidis* in more than one culture with identical results of the antibiotic susceptibility testing makes a contamination less likely. Therefore, we used the results of the antibiotic susceptibility testing in addition to the results of other intraoperative samples as well as the above-mentioned criteria for diagnosing a PJI and analyzed the isolates. In total, in 90.1% of all cases with PJI caused by *S. epidermidis*, the pathogen could be detected in at least two cultures (coagulase-negative staphylococci in total: 80.6%). Further subgroup analysis revealed that in these 80.6% of all cases with PJI caused by coagulase-negative staphylococci and detection in at least two cultures, detection of coagulase-negative staphylococci was possible on average in 3.65 ± 2 of 5.75 ± 2.3 cultures.

Enterococci species were the third most frequent detect pathogen with similar rates in patients with PJI of the hip and knee. In the literature, detection of enterococci in PJI varies between 2.3–15%, which is in accordance with our results of an overall detection rate of 9% [6]. To date, most studies did not detect a significantly higher rate of enterococci in PJI of the hip in comparison to a PJI of the knee [12]. Nevertheless, for treatment, antibiotic susceptibility is essential. Presence of vancomycin-resistant enterococci (VRE) has been reported in only a minority or in case of persisting and often polymicrobial PJIs. The rates of vancomycin-resistance mentioned in literature are up to 12% (of all enterococci), which is consistent with our results as only 2 of 49 isolates displayed a vancomycin-resistance [12,19,20]. Therefore, PJI with vancomycin-resistant enterococci is not to be expected in general. Nevertheless, PJIs with VRE are associated with low rates of infection control and high risk of treatment failure [20,21]. Therefore, knowledge of an expected low or high rate of VRE might be a decisive factor to determine the initial empiric antibiotic therapy. Further studies that evaluate PJI caused by enterococci are needed to define the best possible antimicrobial therapy. 

In contrast to bacterial PJIs, fungal infections are still a rare finding in PJI, which, however, are burdened with huge difficulties in management and eradication [5,22]. In our study fungal infections could be detected in only a minority of the cases (2.4%), while in 30% being part of a polymicrobial PJI (4 of 13 cases). In previous studies, the rate of fungal PJI has been described between 1–2.4% [22,23,24]. Our findings are in accordance to the current literature. Nevertheless, against the background of the challenging and prolonged treatment necessary for eradication if fungal species are detected they should always be treated accordingly. 

Detection of Gram-negative pathogens was possible in 21% of all cases, while in case of a polymicrobial infection they could only be detected in 17%. The detections of different species varied with *E. coli* followed by *P. mirabilis* being the most frequent detected species in all our PJI cases, while *P. mirabilis* and *K. pneumoniae* being the most frequent detected Gram-negative rods in polymicrobial PJI. Interestingly, previous studies reported detection rate of between 6–23% for Gram-negative pathogens while outlining the importance of a correct antibiotic treatment for infect eradication [25]. This is confirmed by our results as resistance to common first-line antibiotics in case of an expected infection with Gram-negative bacteria could be detected for piperacillin-tazobactam in 18.7% of all isolates (ciprofloxacin 9.4%; sulfamethoxazole-trimethoprim 27%). Interestingly, none of the isolates displayed a resistance to meropenem. Therefore, in septic patients with PJI and suspected Gram-negative pathogens, an initial antibiotic therapy with meropenem must be considered. Our data suggest that an empiric antibiotic treatment might lead to treatment failure in case of a Gram-negative PJI, if the causative pathogen is not detected during treatment and antibiotic susceptibility is confirmed or antibiotic treatment—in case of a resistant pathogen—is not changed.

As polymicrobial PJI have been frequently described as a challenge in treatment of PJI, knowledge of the microbiological spectrum might be very helpful. Our most important finding was that *S. epidermidis*, followed by *E. faecalis* and *S. aureus* were the most frequently detected pathogens. In contrast, previous studies reported inconsistent results with describing *S. aureus* (53–54%), followed by coagulase-negative staphylococci (20–21%) and *E. faecalis* (14–15%) as the most common pathogen [5,16], while Flurin et al. reported *S. epidermidis* as the most frequently detected pathogen (60%) in polymicrobial PJI [26]. We could not detect a most frequent combination of pathogens in polymicrobial PJI. No co-pathogen was found more frequently than others, except the combination of *S. aureus* and *E. faecalis* (overall), *E. faecalis* and *P. mirabilis* (hip) and *E. faecalis* and *E. faecium* (knee). As these combinations could only be detected in 3–4 cases, it is not possible to give a final recommendation for antibiotic therapy. *S. epidermidis* as most frequent pathogen in polymicrobial PJI could be detected with a broad combination including streptococci, enterococci or other coagulase-negative staphylococci. Further studies are needed for a better understanding of pathogen combinations in polymicrobial PJI. 

An essential part of successful treatment of PJI is the targeted antibiotic therapy. Today, empirical antibiotic therapy with ampicillin-sulbactam or amoxicillin-clavulanic acid is often recommended as first-line [4]. Nevertheless, recommendations differ between countries, microorganism prevalence and resistance pattern, often leading to the suggestion, that only knowledge of the local microbiological spectrum allows the best possible choice [27]. As all antibiotics may cause side effects and exert selective pressure resulting in increasing resistance rates, the choice always has to be justified. Our results indicate that in our clinic in cases of an unknown pathogen, empiric therapy with vancomycin must be considered and should be given priority over, e.g., amoxicillin-clavulanic acid, because of the rates of oxacillin-resistant *S. epidermidis* isolates. After analysis of our data, we changed our initial empiric therapy from beta-lactam-antibiotics to vancomycin until identification of the causative pathogen. In case of a suspected Gram-negative pathogen, we administer piperacillin/tazobactam in addition until identification of the pathogen. In the rare case of a septic patient with a suspected Gram-negative pathogen, we exchange piperacillin/tazobactam for meropenem. After implantation of the new prosthesis and depending on the detected pathogen, a combination with rifampicin may be chosen for treatment. Nevertheless, it should not be given as a monotherapy or after implantation of a temporary spacer to prevent emerge of rifampicin resistance [4,28]. In summary and according to our data, an empiric combination of vancomycin and piperacillin-tazobactam might be necessary to additionally address Gram-negative bacteria. Nevertheless, and under consideration of comorbidities of the patient, the treating team should always be aware of potential alternatives for vancomycin, such as teicoplanin, daptomycin or linezolid. For final evaluation, further studies are needed. Targeted antibiotic therapy should be used once a causative pathogen has been detected.

Despite the overall results obtained by this study, it surely has some limitations. First, based on its retrospective design, there is a collection and selection bias. We were not able to access previous microbiological results of foreign laboratories or the exact history of the previously performed antibiotic therapy. Therefore, a potential influence cannot be evaluated. Additionally, this study was conducted at a single hospital, which might contribute to a selection bias. Moreover, this hospital is tertiary endoprothetic referral center, where patients are often transferred to due to orthopedic (complicated and prolonged treatment) or non-orthopedic (multimorbidity, potential need for prolonged intensive-care therapy) factors as part of a complicated history of infection. In addition, we only included the first episode of PJI. We did not include pathogens detected in intraoperative samples of subsequent revision surgeries. 

## 5. Conclusions

While the majority of pathogens in cases of PJI of the hip or knee could be identified as coagulase-negative staphylococci (most prevalent *S. epidermidis*), pathogens such as Gram-negative bacteria and fungi still play—in summary—an important role. 

Moreover, effective treatment based on antibiotic susceptibility testing is decisive for treatment success. For initial empirical therapy vancomycin or alternatives might provide the highest antimicrobial coverage in case of an empiric therapy/unknown pathogen. A lack of knowledge of the local microbiological profile might lead to an ineffective therapy with severe consequences for the patient. 

## Figures and Tables

**Table 1:** Demographics of hip and knee periprosthetic joint infections.

Demographic Characteristics	Hip		Knee		*p* Value
Number of patients	250		182		
Male	118 (47.2%)		97 (53.3%)		0.242
Female	132 (52.8%)		85 (46.7%)		
Age [years], Mean ± SD (range)	69.76 ± 12.87 (18.6–97.92)		69.76 ± 10.87 (35.4–89.46)		0.755
BMI [kg/m^2^)	29.86 ± 8.56		30.01 ± 7.73		0.791
Preoperative creatinine [mg/dl]	0.94 ± 0.49		1.15 ± 1.0		0.017
Preoperative C-reactive Protein [mg/dl]					
- Acute early onset	87.05 ± 70.5		87.29 ± 73.56		0.987
- Persisting early onset	77.37 ± 49.19		-		
- Acute late onset	129.46 ± 98.61		141.89 ±122.69		0.630
- Chronic late onset	43.92 ± 64.01		69.96 ± 98.73		0.009
**Comorbidities**					
- Hypertension	212	85.1%	164	90.1%	0.249
- Smoking	68	27.4%	57	31.5%	0.359
- Diabetes mellitus	78	31.5%	78	42.9%	0.015
- Alcoholism	25	10.1%	19	10.4%	0.903
- Cirrhosis	15	6%	20	11%	0.064
- Malignancy	39	15.7%	17	9.3%	0.052
- Rheumatoid arthritis	20	8.1%	22	12.1%	0.162
- Immunosuppression	38	15.3%	44	24.2%	0.021
- Chronic kidney disease	63	25.2%	64	35.2%	0.025
**Time to infection**					
- Acute early onset	53	21.2%	28	15.3%	
- Persisting early onset	3	1.3%	0	0	
- Acute late onset	34	13.5%	45	24.7%	
- Chronic late onset	160	64%	109	60%	

**Table 2:** Microorganisms in cases mono- and polymicrobial polymicrobial periprosthetic joint infection.

Pathogen	Hip	% of Isolates	Knee	% of Isolates	Hip and Knee	% of Isolates
**Aerobic Gram-positive**	241	77.00%	178	79.11%	419	77.88%
- Coagulase-negative *staphylococci*	152	48.56%	88	39.11%	240	44.61%
- *Staphylococcus aureus*	40	12.78%	37	16.44%	77	14.31%
- *Enterococcus faecalis*	22	7.03%	16	7.11%	38	7.06%
- *Enterococcus faecium*	6	1.92%	5	2.22%	11	2.04%
- *Streptococcus species*	15	4.79%	29	12.89%	44	8.18%
- *Micrococcus luteus*	3	0.96%	1	0.44%	4	0.74%
- *Granulicatella adiacens*	1	0.32%	0	0.00%	1	0.19%
- *Kocuria rhizophila*	1	0.32%	0	0.00%	1	0.19%
- *Corynebacterium species*	1	0.32%	2	0.89%	3	0.56%
**Rod-shaped or anaerobic Gram-positive**	19	6.07%	14	6.22%	33	6.13%
- *Cutibacterium acnes*	12	3.83%	12	5.33%	24	4.46%
- *Cutibacterium avidum*	5	1.60%	0	0.00%	5	0.93%
- *Clostridium tertium*	1	0.32%	0	0.00%	1	0.19%
- *Erysipelothrix rhusiopathiae*	1	0.32%	0	0.00%	1	0.19%
- *Pseudarthrobacter sulfonivorans*	0	0.00%	1	0.44%	1	0.19%
- *Peptoniphilus coxii*	0	0.00%	1	0.44%	1	0.19%
**Gram-negative**	43	13.74%	25	7.99%	68	21.73%
- *Escherichia coli*	6	1.92%	9	4.00%	15	2.79%
- *Proteus mirabilis*	8	2.56%	5	2.22%	13	2.42%
- *Proteus vulgaris*	1	0.32%	0	0.00%	1	0.19%
- *Enterobacter cloacae* complex	7	2.24%	0	0.00%	7	1.30%
- *Serratia marcescens*	5	1.60%	1	0.44%	6	1.12%
- *Pseudomonas aeruginosa*	4	1.28%	1	0.44%	5	0.93%
- *Klebsiella pneumoniae*	5	1.60%	6	2.67%	11	2.04%
- *Klebsiella aerogenes*	3	0.96%	1	0.44%	4	0.74%
- *Klebsiella oxytoca*	1	0.32%	1	0.44%	2	0.37%
- *Acinetobacter baumannii* complex	1	0.32%	0	0.00%	1	0.19%
- *Bacteroides vulgatus*	1	0.32%	0	0.00%	1	0.19%
- *Citrobacter koseri*	1	0.32%	0	0.00%	1	0.19%
- *Porphyromonas somerae*	0	0.00%	1	0.44%	1	0.19%
**Fungus**						
- *Candida* species	8	2.56%	5	2.22%	13	2.42%
*Bacillus* spp.	1	0.32%	2	0.89%	3	0.56%
*Brevibacterium luteolum*	1	0.32%	1	0.44%	2	0.37%
Total	313	100.00%	225	100.00%	538	100.00%

**Table 3:** Microorganisms in cases with polymicrobial periprosthetic joint infection.

Pathogen	Hip	% of All Polymicrobial PJI of the Hip (*n* = 51)	Knee	% of All Polymicrobial PJI of the Knee (*n* = 34)	Total	% of All Polymicrobial PJI (*n* = 85)
**Aerobic Gram-positive**	79		57		136	
- *Staphylococcus epidermidis*	28	54.90%	14	41.18%	42	49.41%
- *Enterococcus faecalis*	12	23.53%	8	23.53%	20	23.53%
- *Staphylococcus aureus*	10	19.61%	9	26.47%	19	22.35%
- *Enterococcus faecium*	4	7.84%	5	14.71%	9	10.59%
- *Staphylococcus haemolyticus*	7	13.73%	1	2.94%	8	9.41%
- *Staphylococcus capitis*	7	13.73%	1	2.94%	8	9.41%
- *Staphylococcus lugdunensis*	2	3.92%	4	11.76%	6	7.06%
- *Staphylococcus warneri*	1	1.96%	1	2.94%	2	2.35%
- *Staphylococcus hominis*	2	3.92%	3	8.82%	5	5.88%
- *Streptococcus agalactiae*	1	1.96%	5	14.71%	6	7.06%
- *Streptococcus mitis/oralis*	1	1.96%	3	8.82%	4	4.71%
- *Streptococcus pneumoniae*	0	0.00%	1	2.94%	1	1.18%
- *Streptococcus infantarius subspecies coli*	1	1.96%	0	0.00%	1	1.18%
- *Streptococcus gordonii*	0	0.00%	1	2.94%	1	1.18%
- *Streptococcus gallolyticus*	0	0.00%	1	2.94%	1	1.18%
- *Streptococcus dysgalactiae*	1	1.96%	0	0.00%	1	1.18%
- *Granulicatella adiacens*	1	1.96%	0	0.00%	1	1.18%
- *Corynebacterium afermentans*	1	1.96%	0	0.00%	1	1.18%
**Rod-shaped or anaerobic Gram-positive**	10		4		14	
- *Cutibacterium acnes*	4	7.84%	3	8.82%	7	8.24%
- *Cutibacterium avidum*	5	9.80%	0	0.00%	5	5.88%
- *Peptoniphilus coxii*	0	0.00%	1	2.94%	1	1.18%
- *Clostridium tertium*	1	1.96%	0	0.00%	1	1.18%
**Gram-negative**	20		13		33	
- *Proteus mirabilis*	5	9.80%	2	5.88%	7	8.24%
- *Klebsiella pneumoniae*	2	3.92%	4	11.76%	6	7.06%
- *Klebsiella aerogenes*	3	5.88%	1	2.94%	4	4.71%
- *Escherichia coli*	2	3.92%	3	8.82%	5	5.88%
- *Pseudomonas aeruginosa*	2	3.92%	1	2.94%	3	3.53%
- *Enterobacter cloacae complex*	3	5.88%	0	0.00%	3	3.53%
- *Serratia marcescens*	1	1.96%	1	2.94%	2	2.35%
- *Porphyromonas somerae*	0	0.00%	1	2.94%	1	1.18%
- *Bacteroides vulgatus*	1	1.96%	0	0.00%	1	1.18%
- *Acinetobacter baumannii* complex	1	1.96%	0	0.00%	1	1.18%
**Fungus**	3		1		4	
- *Candida albicans*	2	3.92%	1	2.94%	3	3.53%
- *Candida tropicalis*	1	1.96%	0	0.00%	1	1.18%
*Bacillus* species	1	1.96%	1	2.94%	2	2.35%
Total	113		76		189	

**Table 4:** Antibiotic resistance of selected pathogens against selected antibiotics.

Pathogen		Hip		Knee		Total		Total
		r	s	r	s	r	s	
*S. aureus*	oxacillin	4	35	1	36	5	71	77 ^a^
	rifampicin	3	36	2	35	5	71	77 ^a^
Coagulase negative staphylococci	oxacillin	85	64	57	28	142	92	234 ^b^
	rifampicin	40	111	21	66	61	177	238 ^c^
	vancomycin	0	150	0	86	0	236	240 ^d^
*Enterococcus* species	ampicillin	6	22	5	16	11	38	49
	vancomycin	2	26	0	21	2	47	49
Gram-negative ^g^	piperacillin-tazobactam	11	29	3	21	12	52	64 ^e^
	ciprofloxacin	2	38	4	20	6	58	64 ^e^
	sulfamethoxazole-trimethoprim	8	28	8	15	16	43	59 ^f^
	meropenem	0	43	0	25	0	68	68

r = resistant; s = sensitive; ^a^: susceptibility testing according to EUCAST for one isolate not possible; ^b^: susceptibility testing according to EUCAST for six isolates not possible; ^c^: susceptibility testing according to EUCAST for two isolates not possible; ^d^: susceptibility testing according to EUCAST for four isolates not possible. ^e^: without *A. baumanii* complex, *B. vulgatus*, *P. somerae*, *P. vulgaris;* ^f^*:* without *A. baumanii* complex, *B. vulgatus*, *P. somerae*, *P. vulgaris* and *P. aruginosa;* ^g^*:* including non-fermenter: *P. aeruginosa* (5) and *A. baumannii* complex (1).

## Data Availability

The data presented in this study are available in the manuscript.
